# Optimal Parameters to Milk Murciano-Granadina Goats in Mid and Low-Line Milking Parlours

**DOI:** 10.3390/ani13071155

**Published:** 2023-03-24

**Authors:** Joel Bueso-Ródenas, Gema Romero, Amparo Roca, Francisco Moya, Manuel Alejandro, José Ramón Díaz

**Affiliations:** 1 Departamento Producción Animal y Salud Pública, Universidad Católica de Valencia (UCV), C/Guillem de Castro 94, 46001 Valencia, Spain; joel.bueso@ucv.es; 2 Departamento Tecnología Agroalimentaria, Universidad Miguel Hernández (UMH), Ctra. de Beniel km 3.2, 03312 Orihuela, Spain; gemaromero@umh.es (G.R.); aroca@umh.es (A.R.);; 3 C/Anabel Segura, 7, 28108 Madrid, Spain

**Keywords:** dairy goats, somatic cell count, milking efficiency, milk cortisol

## Abstract

**Simple Summary:**

There were not long-term studies in the literature reviewed on how machine milking parameters (pulsation rate, pulsation ratio and system vacuum) affect milk production, milk composition, animal welfare and udder health throughout the lactation of Murciano-Granadina goats. After previous experiments developed in short term by this research group, the aim of the present experiments was to test during one entire lactation duration the best combination of system vacuum and pulsation parameters using two types of milking parlours, one with low-line pipes and the other with mid-line pipes to obtain optimal values of milking duration, milk yield, animal welfare and udder health. The results confirmed that the optimal parameters to milk Murciano-Granadina goats are different in mid-line milking parlours and in low-line milking parlours. The milking parameters must be correctly programmed to achieve optimal milking efficiency and milk quality values, enhancing the animals’ sanitary status and farm profitability. The conclusions of this project have established work guidelines for small ruminants’ farmers and technicians to improve milk quantity, milk quality and animal welfare.

**Abstract:**

Recent short-term studies on Murciano-Granadina goats have established that the optimal parameters to set up the milking machines are different according to the milk pipes height. Two groups of 52 fresh goats each were employed in 2 different experiments to confirm during an entire lactation period the best combinations of system vacuum pulsation rate and pulsator ratio in low-line and mid-line milking parlours. The experiment performed in the low-line milking parlour included one group milked with 40 KPa vacuum system level, 90 puls/min pulsation rate and 60/40 pulsation and a second group milked with 38 KPa vacuum system level, 90 puls/min pulsation rate and 60/40 pulsation ratio. The experiment carried out in mid-line included one group milked with 40 KPa vacuum system level, 90 puls/min pulsation rate and 60/40 pulsation ratio and a second group milked with 40 KPa vacuum system level, 120 puls/min pulsation rate and 60/40 pulsation ratio. Variables studied included milking efficiency, milk composition, cortisol, SCC and intramammary infections, teat-end oedema after milking and vacuum dynamics during milking. Considering the results of an entire lactation period, it was confirmed that when milking in mid-line, the combination of 40 KPa system vacuum, 90 cycles/min pulsation rate and 60/40 pulsator ratio showed optimal results of the above-mentioned variables. On the other hand, the use of 40 KPa in a low-line system increased the milk cortisol values (0.34 ± 0.1 vs. 0.44 ± 0.1 ng/mL) without any other advantage. Thus, the recommendation is to use a combination of 38 KPa system vacuum, 90 cycles/min pulsation rate and 60/40 pulsator ratio to enhance animal welfare.

## 1. Introduction

In recent decades, machine milking in dairy goat farms has resulted in a useful tool to reduce the working times and improve milk quantity and quality. Research on machine milking of dairy goats, including milking performance [1], milking routines [2], milking frequency and udder compartmentalisation in different breeds has been carried out [3]. A typical milking parlour design for goats is side by side parlours [4,5,6]. Manufacturers have recommended different parlour designs according to the farmer’s needs. The characteristics of a milking machine design include the number of animals fitting in the parlour and the number of milking units. Potentially, the larger the number of animals fitting in the milking parlour and the animals being milked at the same time, the shorter the time employed in milking the herd will be. Another issue to consider is the milk pipes height. The use of low-line milking parlours allows for having one milking unit per animal in each platform, which will shorten the time employed to milk the entire flock. Moreover, low-line milking machines ensure higher vacuum stability in the short milk tubes [7] and potentially lower milk lipolysis [8]. However, mid- and high-line milking parlours offer better performance of the parlour expressed in animals milked per milking unit, as these systems let the operator turn the milking unit to both sides of the parlour, reducing the time in which the milking unit is not being used. Moreover, mid- and high-line milking parlours make the installation of milk meters or automatic cluster removers easier [4]. In addition, several studies carried out in small ruminants have discarded the traditional idea in which mid-line milking parlours were related to poorer udder health [8,9]. The choice between low-line or mid-line milking systems is not easy for farmers and often depends on the farmer’s personality or the specific needs of each farm. In addition to these characteristics of the milking machines, there are some parameters that will affect the milking performance of the parlour, as they affect the individual milking of each goat. Some of them are related to the characteristics of the animal. These characteristics are both anatomical and physiological and include position and angles of the teats, the teat sphincter strength as well as the animal’s capacity to release residual milk under the oxytocin stimulus [5,6]. Other parameters are basically mechanical, including the system vacuum level of the milking machine and the pulsation rate and ratio [1,10,11]. 

With the aim of finding a balance between milking performance, udder health and animal welfare, some experiments have been carried out in different breeds of goats. Fernández et al. [5] carried out an experiment in Murciano-Granadina goats in a mid-line milking parlour and found better milking efficiency, reducing milking duration, when combining 42 KPa system vacuum and 120 cycles/min of pulsation rate than when 40 KPa system vacuum and 90 cycles/min pulsation rate were combined. In a similar experiment, Fernández et al. [10], again using a mid-line milking parlour, found that the use of high vacuum level (44 KPa compared to 42 KPa) entailed higher values of the average milk flow. Despite this fact, the use of 44 KPa entailed higher teat congestion after milking and showed higher values in the milk electrical conductivity at the end of lactation, which could be an indication of long-term damage to udder health. Considering the hypothesis that the parameters to set up the milking machine should be adapted to the height of the milk line, this research group performed two experiments with Murciano-Granadina goats in both milk line heights [11]. The aim was to establish the optimal combination of system vacuum level (36 or 40 KPa), pulsator ratio (50/50 or 60/40) and pulsation rate (90 or 120 pulse/min) that would reach the best values for milking fractioning (relation between machine milk, hand stripping milk and residual milk), milking dynamics (milk flows), animal welfare and mammary gland sanitary status. In mid-level pipe milking parlours, a combination of 40 KPa system vacuum, 60/40 pulsator ratio and 90 or 120 cycles/min pulsation rate achieved optimal machine milk fraction and milking duration values without negative effects on the health status of the animals. Additionally, in low-level pipe milking parlours, when 36 KPa system vacuum, 60/40 pulsator ratio and 90 or 120 cycles/min pulsation rate were compared to other combinations with 40 KPa system vacuum, machine milk fraction values were maintained and preserved the teat-end status after milking and the milk cortisol level. However, this combination slightly achieved worse milk flow values. It was concluded that it was necessary to test, in low-level pipes milking parlours, if the use of 38 KPa could achieve optimal milk flows during milking without negative effects on the teat-end status and the animal welfare. Moreover, it was concluded that additional long-term experiments should be performed to confirm these results and to establish the optimal parameters to milk Murciano-Granadina goats, considering their effects on milk yield, milk composition and sanitary status of the mammary gland at both milk line heights. The aim of the present study was to find the optimal milking parameters for the milking of Murciano-Granadina goats during one lactation in terms of milk yield, milking duration, milk composition, animal welfare, mammary gland health status (somatic cell count and frequency of mastitis), teat-end status and vacuum dynamics during milking, comparing the combinations that obtained the best results in the previous short term experiment [11]: in low line (i) 40 KPa vacuum system level, 90 puls/min pulsation rate and 60/40 pulsation ratio vs. (ii) 38 KPa vacuum system level, 90 puls/min pulsation rate and 60/40 pulsation ratio and, in mid-line, (i) 40 KPa vacuum system level, 90 puls/min pulsation rate and 60/40 pulsation ratio vs. (ii) 40 KPa vacuum system level, 120 puls/min pulsation rate and 60/40 pulsation ratio. 

## 2. Materials and Methods

### 2.1. Facilities and Animal Handling

Two experiments were carried out using the Murciano-Granadina goat flock of the research farm of the Miguel Hernández de Elche University (UMH). The farm facilities included a free-stall barn (1.5 square metres of cereal straw litter per animal) where goats also had access to outdoor space (3 square metres per animal). The goats were fed ad libitum with cereal straw and alfalfa hay and cereal mixture according their days in milk. The reproductive rhythm of the farm was 1 parturition per year, including 10 months of lactation and 2 months of dry period. After birth, the kids were separated from the mother so that the feeding of the kids was through artificial milk in a different barn. The goats were mechanically milked once a day at 8:00 in the morning. The milking parlour included 2 platforms with a low-line milking system (1 × 12 × 12) and also a mid-line milking system (2 × 12 × 12) set up 120 cm above the milking platform. The equipment included electronic pulsators (StimoPuls Apex M, GeaFarm Technologies, Bönen, Germany) which allowed us to vary the pulsation parameters and also two vacuum regulators to easily vary the system vacuum level. Additionally, both systems included automatic cluster removers that were programmed to detach the clusters after 60 s of minimum milking duration and a combination of 150 g/min milk flow threshold and a delay time of 10 s [12]. The milking routine applied did not include udder stimulation but included cluster attachment without delay after animal positioning on the platform, machine milking and end of machine milking through automatic cluster removal. Under the judgement of the experienced milkers, if the extraction of milk from the udder was uncomplete after automatic cluster remover activation, then the milker rapidly performed a double cluster attachment (DCA), brief and careful machine stripping, manual vacuum shut off and manual removal of the cluster. After milking, teat-dipping was carried out in an iodine solution. 

### 2.2. Experimental Design

#### 2.2.1. Pre-Experimental Period

The pre-experimental period lasted one month. To determine the initial conditions of the goats and then perform a selection of the animals involved in the experiments, a first sampling was performed in 114 goats of the same parturition lot (3 ± 1 postpartum weeks). These samplings included milk yield, milking duration (see Section 2.3.1) and mammary gland health status (see Section 2.3.2). From the results of these variables, goats free of clinical mastitis, whose milking duration was less than 6 min and milk yield was higher than 0.85 kg were selected for the experiment. Moreover, animals suffering clinical mastitis or asymmetries in the mammary glands due to previous episodes of mastitis were discarded for the experiments. From this point, 104 goats were selected and clustered into 4 different groups of 26 animals according to their number of lactations (age), milking duration, machine milk yield, and mammary gland health status. Two of these groups (52 goats) were used to carry out one experiment using the low-line milking machine and the other two groups (other 52 goats) were employed in the other experiment using the mid-line milking machine. Later, during this pre-experimental period and to consider values of the studied variables as covariables in the statistical analyses, samplings regarding milking efficiency and milk cortisol (see Section 2.3.1), mammary gland health status (see Section 2.3.2), teat-end status (see Section 2.3.3) and vacuum level variables (see Section 2.3.4) were carried out on non-consecutive days (Table 1). During the pre-experimental period, at both milk line heights, the milking machine was set up with 40 KPa vacuum system level, 90 puls/min pulsation rate and 60/40 pulsation ratio. 

#### 2.2.2. Experimental Period 

Both experimental periods lasted 7 months. The experiment performed in the low-line milking parlour included one group that was milked with 40 KPa vacuum system level, 90 puls/min pulsation rate and 60/40 pulsation ratio (LL-40KPa-90ppm-60/40) and a second group that was milked with 38 KPa vacuum system level, 90 puls/min pulsation rate and 60/40 pulsation ratio (LL-38KPa-90ppm-60/40). The experiment carried out in mid-line included one group that was milked with 40 KPa vacuum system level, 90 puls/min pulsation rate and 60/40 pulsation ratio (ML-40KPa-90ppm-60/40) and a second group that was milked with 40 KPa vacuum system level, 120 puls/min pulsation rate and 60/40 pulsation ratio (ML-40KPa-120ppm-60/40).

The four groups of animals (two groups per experiment) followed the same milking routine during the pre-experimental and experimental periods.

### 2.3. Samplings and Variables Analysed

Similar to what was performed in the pre-experimental period, during the experimental period, 4 samplings were performed every month. These 4 samplings included: (i) milking efficiency, milk composition and milk cortisol; (ii) mammary gland health status; (iii) teat-end status and (iv) vacuum dynamics during milking. The samplings always took place on non-consecutive days.

#### 2.3.1. Milking Efficiency, Milk Composition and Milk Cortisol

For the milking efficiency tests, Lactocorder^®^ devices (Lactocorder, Balgach, Switzerland) were installed in each individual milking unit. Thus, milking duration (MD, s), machine milk yield (MMY, kg), average milk flow (AMF, kg/min) and peak flow rate (PFR, kg/min) during machine milking were recorded. In the case of a DCA, this was registered and the machine milk was extracted during this machine stripping (Kg). After machine milking, rapid manual extraction and weighing (digital scale of ± 1 g precision Zanussi^®^, Alcobendas, Spain) of hand-stripping milk (HSM, g) was carried out. Then, 4 IU of oxytocin (Facilpart^®^, Syva, León, Spain) was administered in the jugular vein of the goats to extract and weigh the residual milk (RM, g) similarly to what was performed with the HSM variable. Total milk (TM, Kg) was calculated as the sum of MM, HSM and RM.

Additionally, during this sampling, the Lactocorder devices were set up to take, throughout the machine milking, samples of 50 mL of milk from each goat. A MilkoScan FT 120 device (Foss, Hillerød, Denmark) was used to determine the lactose (L, %), protein (P, %) and fat (F, %) content. Additionally, from the same milk samples, 10 mL aliquots of milk was collected and cooled to 4 °C and centrifuged at 2000× *g* for 20 min to remove the milk fat. Next, 10 μL of azidiol was added as a preservative, and the samples were sent to the Endocrinology Laboratory of the Complutense University of Madrid (Spain). The extraction of cortisol was performed according to the procedure described by Hagen et al. [13]. The subsequent determination of milk cortisol concentrations (ng/mL) was performed by enzyme immunoassay [14,15]. 

#### 2.3.2. Mammary Gland Health Status

From the 104 goats enrolled in both experiments, milk sampling of each teat was performed to develop somatic cell counts and microbiological cultures. The milk sampling was performed manually before milking cluster attachment. The procedure included forestripping and cleaning with individual sterile gauzes impregnated in 70% ethanol. This was followed by a sampling of 50 mL of milk for the somatic cell counts. Then, another cleaning procedure was performed and another 5 mL of milk was collected for the bacteriological cultures. Somatic cell count milk samples per gland were analysed using a Fossomatic 5000 device (Foss, Hillerød, Denmark) in LICOVAL (Interprofessional Dairy Laboratory, Valencia, Spain). For the microbiological cultures, 20 μL of milk from the other 5 mL samples were seeded onto 5% sheep blood agar plates. The incubation was carried out at 37 °C and examined at 48 h. It was established that a mammary gland whose culture plates had more than 5 similar colonies and/or in which somatic cell counts were above 1.5 × 10^6^ somatic cells/mL was suffering subclinical mastitis. This procedure was also followed once a month during the experimental period. Frequency of animals suffering subclinical mastitis was registered for the four groups of animals. Additionally, after the milk composition analyses (see Section 2.3.1), SCC per udder was analysed following the same method as in SCC per gland. 

#### 2.3.3. Teat-End Status

Scans of the goats’ teats were performed before and after the machine milking with an ultrasonography device (Agroscan AL, ECM, Noveko International Inc., Angoulême, France) according to the procedures of Díaz et al. [16]. The resulting images were analysed using a specific software (ECOPEZÓN Miguel Hernández University, Elche, Spain) following the process reported by Alejandro et al. [17] to obtain the values of the following variables: (i) increase in teat wall thickness (ITWT, %), (ii) increase in teat wall area (ITWA, %) and (iii) increase in teat-end wall area (ITEWA, %).

#### 2.3.4. Vacuum Level Variables

During the experimental period, every month, the milking of 12 random animals per treatment was analysed using a Pulsotest Comfort (GeaFarm Technologies, Bönen, Germany). This device was connected to the short milk tube to perform dynamic vacuum levels tests. The vacuum level was measured during 60 s of the main milking phase when milk was visualised flowing from both teats through both short milk tubes. The cited device recorded the minimum vacuum level (KPa), maximum vacuum level (KPa) and average vacuum level (KPa) during the 60 s measurement period. Additionally, the difference between minimum and maximum vacuum level was calculated as Vacuum drop (KPa).

### 2.4. Statistical Analysis

The SCC values per udder were transformed (LSCC = log10 SCC) with the aim of achieving normality of their distribution [18].

A general linear model (Proc GLM, SAS 9.2., 2012) was applied to calculate the values of MMY, MD, HSM, RM, TM, fat, protein, milk cortisol, ITWT, ITWA and ITEWA variables during the pre-experimental period. The group of animals (1 to 4) was considered as a factor.

To determine the effect of the treatment in both experiments performed (experiment in low-line: LL-40KPa-90ppm-60/40 and LL-38KPa-90ppm-60/40; experiment in mid-line: ML-40KPa-90ppm-60/40 and ML-40KPa-120ppm-60/40) on the MMY, MD, HSM, RM, TM, LSCC, fat, protein, milk cortisol, ITWT, ITWA and ITEWA variables, a linear mixed model (Proc GLIMMIX, SAS 9.2., 2012) was used. Additionally to the treatment employed, the considered fixed effects included the month of lactation (controls 1 to 7). The interaction between the treatment and the month of lactation was not significant and not included in the model. Moreover, in this model, the values of all variables recorded during the pre-experimental period were added as covariable. Specifically, in the MD variable, the MM values were also treated as a covariable. Finally, in this model, to consider repeated samplings throughout lactation in the same goat, this was considered a random term and a “compound symmetry” covariance structure was applied. 

In both experiments carried out, 2 Chi-square analyses (Proc Freq. SAS 9.2., 2012) were performed to study the association of the number of new cases of mastitis with the treatments applied (experiment in low-line: LL-40KPa-90ppm-60/40 and LL-38KPa-90ppm-60/40; experiment in mid-line: ML-40KPa-90ppm-60/40 and ML-40KPa-120ppm-60/40).

Finally, a general linear model (Proc GLM, SAS 9.2., 2012) was applied to study the association of each treatment (experiment in low-line: LL-40KPa-90ppm-60/40 and LL-38KPa-90ppm-60/40; experiment in mid-line: ML-40KPa-90ppm-60/40 and ML-40KPa-120ppm-60/40) with the maximum vacuum level, average vacuum level and vacuum drop.

## 3. Results

Results of the variables studied during the pre-experimental period related to milking fractioning (MMY, HSM and RM), milking kinetics (MD, PFR and AMF), mammary gland health status (number of goats with subclinical mastitis and somatic cell count), teat-end status (ITWT, ITWA, ITEWA), milk compositions (fat, protein and lactose) and animal welfare (milk cortisol) showed no differences between the two experimental groups of animals (Table 1). For both experiments, the month of lactation (controls 1 to 7) had effect on the variables MMY, RM, TM, MD, AMF, fat, protein and SCC per udder. MMY, RM, TM, MD and AMF decreased at each control as lactation progressed. The fat values were higher at controls 1 and 7; the protein values were higher at controls 1, 2 and 7 and SCC per udder showed higher values as lactation progressed. On the other hand, there was no effect on HSM, milk cortisol, teat end status variables and vacuum level variables which remained stable during the entire lactation.

### 3.1. Low-Line Experiment

In the experiment carried out in low-line, MMY and HSM variables showed no differences. Despite this, the RM values showed differences between LL-40KPa-90ppm-60/40 and LL-38KPa-90ppm-60/40. Thus, the use of 40 KPa improved milking fractioning with a difference of 39 g in this variable. This result was not related to changes in the TM variable, which remained similar in both treatments tested. Regarding the milking kinetics, there were no differences between treatments either, as the MD, PFR and AMF variables showed similar values. The treatment had no effect on the milking routine, as the frequency of DCA was similar in both groups of animals (10.6% in LL-40KPa-90ppm-60/40 vs. 9.2% in LL-38KPa-90ppm-60/40). Nevertheless, when a DCA was needed, the milking duration was higher in LL-40KPa-90ppm-60/40 (216 ± 22 s) than in LL-38KPa-90ppm-60/40 (159 ± 22 s). The machine milk values recorded during this DCA followed the same trend, being greater in LL-40KPa-90ppm-60/40 (0.25 ± 0.03 kg) than in LL-38KPa-90ppm-60/40 (0.14 ± 0.05 s). In the same experiment, the teat-end status variables (ITWT%, ITWA% and ITEWA%) were not affected by the treatment employed. The mammary gland health status, in terms of somatic cell count, new cases of mastitis and the milk composition (fat, protein and lactose) did not show differences between treatments. However, the milk cortisol variable of the animals following the LL-38KPa-90ppm-60/40 treatment showed lower values than those of LL-40KPa-90ppm-60/40 (0.34 ± 0.1 vs. 0.44 ± 0.1 ng/mL, respectively, Table 2). 

Regarding the variables studied in the dynamic vacuum levels tests, as expected, the maximum vacuum level and average vacuum level variables were higher in the animals milked under the LL-40KPa-90ppm-60/40 treatment. Despite this, the vacuum drop during milking was low and similar in both treatments (5.6 KPa in LL-38KPa-90ppm-60/40 and 4.1 in LL-40KPa-90ppm-60/40; Table 2).

### 3.2. Mid-Line Experiments

In the experiments carried out in mid-line (treatments ML-40KPa-90ppm-60/40 and ML-40KPa-120ppm-60/40) there were no differences in any variables related to milking fractioning (MMY, HSM and RM), which entailed the absence of differences in the variable TM. Regarding milking kinetics (MD, PFR and AMF), there were no differences between treatments (Table 3).

Similar to the experiments performed in low-line, the performance of a DCA showed similar frequencies in both treatments. Again, the milking duration of the animals that received a DCA was different in both groups. In this case, the ML-40KPa-90ppm-60/40 treatment showed higher values (198 ± 22 s) of this variable than ML-40KPa-120ppm-60/40 (273 ± 20 s). This second cluster attachment entailed machine milk values of 0.11 ±0.03 kg in the case of ML-40KPa-90ppm-60/40 and of 0.35 ± 0.02 kg in the case of ML-40KPa-120ppm-60/40. ITWT%, ITWA% and ITEWA% in the experiment performed in mid-line showed no differences between treatments. The milk somatic cell counts (LogSCC), new cases of mastitis milk composition (fat, protein and lactose) and milk cortisol were similar in both treatments (Table 3).

## 4. Discussion

Average values of the variables studied in both experiments are consistent with what is to be expected from this dairy goat breed in the conditions applied [19]. This observation particularly includes the notable good sanitary status of the mammary glands, related to the high relation between primiparous goats and multiparous goats which entailed very low somatic cell count values [20].

Values of the variables showed a variation from the beginning to the end of lactation following the typical pattern of the Murciano-Granadina goats. Lower values of machine milk, residual milk and milking duration were observed as lactation progressed. Values of hand-stripping milk remained constant, as seen in other long-term studies of this breed [19]. The fat and protein values were high at the onset of lactation, becoming lower as lactation continued and high again at the end of lactation as a concentration phenomenon [21]. SCC per udder showed higher values as lactation progressed as it has been observed in other experiments, an aspect that cannot be linked to a worsening of the sanitary status of the mammary glands [20]. In any case, as observed in the previous short term experiment [11], the treatments employed in these experiments did not involve risks for the sanitary status of the mammary glands.

In view of the results, it is reasonable to think, as mentioned in previous studies [4], that the programming of the machine should be different if it is a mid- or a low-line height, since the differences between the system vacuum and the average vacuum at the short milk tubes were between 4.2 and 4.8 KPa in the high-line and from 0.5 to 1.5 in the case of the low-line.

In the low-line experiment and in previous studies [11], the use of a higher system vacuum has been shown to entail a reduction in the residual milk values. This reduction has little practical importance (39 g) in terms of farm profitability and has not affected the milk synthesis by the goats in the long term, but it shows a relation between the system vacuum and its capacity to release milk from the alveolar part of the gland to the cisternal part. Additional studies are needed to establish whether this phenomenon is related to a more physical effect than a physiological one because of a higher oxytocin release. In any case, as seen in previous studies, the milk cortisol was higher in animals milked with higher system vacuum, and it is questionable to establish that this reduction of residual milk could be related to higher animal welfare during the machine milking.

Considering the results shown in the mid-level milking parlour experiment and those from previous studies [19], it can be established that the use in the long term of 40 KPa system vacuum, 90 to 120 cycles/min pulsation rate and 60/40 pulsation ratio achieves optimal values of milking efficiency, teat-end status, mammary gland health status and milk composition. In those previous experiments [19], it was already shown that the use of lower system vacuum (36 KPa) or pulsation ratio (50/50) values entailed an elevation of the milking duration and a worsening of the values of milking fractioning and milking kinetics. This reduction in milking efficiency would potentially affect the profitability of the farm [22]. The use of 120 cycles/minute did not involve any advantage in any variable as shown in other experiments using a higher vacuum system level [5]. In any case, the use of higher system vacuum level potentially causes a worsening of the teat condition and should also be discarded to avoid stress on the animal (as milk cortisol has shown in previous studies). In any case, having no differences when using 40 KPa system vacuum, the use of 90 cycles/min pulsation rate would be a better choice than the use of 120 cycles/min in order to avoid unnecessary wear and tear on the pulsators. 

In previous experiments carried out in a low-level milking parlour [19], it was shown that the use in the short term of 40 KPa system vacuum achieved optimal results for milking fractioning and milking kinetics when it was combined with a 60/40 pulsator ratio and 90 cycles/min pulsation rate. In this experiment, the same combination with 38 KPa, despite showing greater residual milk values, has achieved similar values of the machine milk and milking duration variables which, definitely, are the factors involved in the milking efficiency and, so, in farm profitability. Thus, based on the results of the present study, the only reason to use 40 KPa instead of 38 KPa would be to extract an extra scarce fraction of residual milk (39 g in this experiment). In any case, this questionable benefit would involve effects on the animal welfare, as observed in the results for the milk cortisol variable throughout the lactation.

Regarding the teat-end status, the elevation of system vacuum from 38 to 40 KPa did not involve a higher level of oedema. Nevertheless, a positive relation between system vacuum level and short-term alterations of the teat-end status has been shown in other experiments in goats. These alterations have been found from 4 KPa in low-line milking machines [19] and from 2 KPa in mid-line milking machines [10]. In any case, in our experiment the use of automatic cluster removers and the correct handling during machine strippings by experienced milkers could have helped to palliate the possible effects caused by the difference in system vacuum between both groups of animals. To date, the conditions that developed higher teat-end oedema in small ruminants were higher system vacuum [10], milking without automatic cluster removers [12,22], overmilking [23] or milking with old or liners not adapted to the species [24]. 

In both experiments, the frequency of use of DCA was relatively low in the four groups of animals studied (from 16.2 to 21.9%). Although this is a common operation in small ruminants milking [22,25], it required the complete dedication of an operator to extract milk from only one animal while other animals on the same platform had already been milked and were waiting to exit the milking parlour, delaying the entrance of animals to be milked. Moreover, the results showed that the use of a DCA with machine stripping entailed a considerable increase in the milking duration (from 40 to 81 s on average depending on the treatment) to extract a negligible amount of milk (0.1 to 0.35 kg of milk on average depending on the treatment). In this sense, the strategy to improve the milking efficiency could involve the elimination of this part of the milking routine. This suggestion has previously been put forward in some studies in sheep and goat [26] and could be based on a progressive programme of elimination of animals with undesirable udder morphology or slow milkings [2,6], scheduling a maximum milking duration [27] or clustering in the same milking group those animals with slow milking or those whose morphologies make a DCA mandatory [28]. 

## 5. Conclusions

Considering the results of the present and previous studies, when milking in mid-line pipe milking parlours, it can be concluded that the use of 120 cycles/min pulsation rate did not offer any advantage, and its use could be questionable in terms of the durability of the pulsators. In mid-line pipe milking parlours, the use of 40 KPa of system vacuum combined with 60/40 pulsator ratio and 90 cycles/min pulsation rate is recommended to achieve optimal values of milking efficiency, teat-end status, mammary gland health status and milk composition. On the other hand, the use of 40 KPa in a low-line milking system would be unnecessary and can entail a reduction of animal welfare, as evidenced by the milk cortisol values. In this sense, in low-line milking parlours, it is recommended a combination of 38 KPa of system vacuum combined with 60/40 pulsator ratio and 90 cycles/min pulsation rate to achieve optimal results of milking efficiency, teat-end status, mammary gland health status and milk composition.

## Figures and Tables

**Table 1:** Characteristics of the four groups of goats included at the beginning of the experiments (means ± standard error).

Variable	Group 1 (LL-40-90-60)	Group 2 (LL-38-90-60)	Group 3 (ML-40-90-60)	Group 4 (ML-40-120-60)	SEM	SL
Goats per group (n)	26	26	26	26		
Primiparous goats (n)	10	10	10	11		
Multiparous goats (n)	16	16	16	15		
Goats withsubclinical mastitis (n)	8	9	11	9		
Machine Milk Yield (Kg)	1.94	2.02	2.03	1.96	0.19	ns
Hand-Stripping Milk (g)	232	257	222	201	32	ns
Residual Milk (g)	207 a	224 a	154 b	152 b	9	<0.01
Total Milk (Kg)	2.38	2.49	2.42	2.31	0.2	ns
Milking Duration (s)	203	185	197	220	22	ns
PFR (Kg/min)	1.2	1.2	1.2	1.3	0.1	ns
AvgFlow (Kg/min)	0.71	0.72	0.78	0.76	0.04	ns
ITWT (%)	44.5	53.1	57.8	54.7	6.5	ns
ITWA (%)	94.2	81.5	81.9	74.4	16.6	ns
ITEWA (%)	28.3	28.7	28.7	25.7	4.4	ns
LogSCC (SCC × 1000)	2.26 (181)	2.13 (137)	2.33 (213)	2.14 (138)	0.09	ns
Fat (%)	5.71	5.75	5.76	5.85	0.11	ns
Protein (%)	3.67	3.66	3.53	3.65	0.06	ns
Lactose (%)	4.45	4.51	4.47	4.46	0.03	ns
Milk Cortisol (ng/mL)	0.41	0.44	0.44	0.49	0.08	ns

PFR: peak flow rate; AvgFlow: average milk flow; ITWT: increase in teat wall thickness; ITWA: increase in teat wall area; ITEWA: increase in teat-end wall area; LogSCC: Log_10_ of the somatic cell count values; F: fat; P: protein; L: lactose; ns: not significant. Values in the same row with different letters (a, b) differ at *p* < 0.05.

**Table 2:** Experiment developed in low-lines pipes milking system: results of the variables studied (least square means ± standard error of the mean) according to the different combinations of system vacuum level and pulsation parameters.

Variable	LL-40KPa-90ppm-60/40	LL-38KPa-90ppm-60/40	SEM	SL
Machine Milk Yield (Kg)	1.76	1.74	0.07	ns
Hand-Stripping Milk (g)	185	192	17	ns
Residual Milk (g)	76 a	115 b	9	<0.01
Total Milk (Kg)	2.02	2.05	0.07	ns
Milking Duration (s)	161	172	8	ns
PFR (Kg/min)	1.1	1.1	0.06	ns
AvgFlow (Kg/min)	0.65	0.61	0.03	ns
nDCA (%)	34 (18.9)	29 (16.2)		ns
MD (DCA)	206 a	259 b	21	<0.01
ITWT (%)	51.1	47.5	3.1	ns
ITWA (%)	54.5	54.7	5.7	ns
ITEWA (%)	21.2	20.1	1.7	ns
LogSCC (SCC × 1000)	2.46 (288)	2.34 (218)	0.09	ns
MASTNC	6	7		ns
Fat	5.13	5.13	0.11	ns
Protein	3.52	3.50	0.05	ns
Lactose	4.16	4.14	0.02	ns
Milk Cortisol (ng/mL)	0.44 a	0.34 b	0.04	<0.01
MaxVL (KPa)	40.5 a	38.2 b	0.1	<0.01
AvgVL (KPa)	39.5 a	36.5 b	0.3	<0.01
Vacuum Drop (KPa)	4.1	5.6	1.1	ns

PFR: peak flow rate; AvgFlow: average milk flow; nDCA: frequency of double cluster attachment; MD (DCA): milking duration in case of a double cluster attachment; ITWT: increase in teat wall thickness; ITEWA: increase in teat-end wall area; ITWA: increase in teat wall area; LogSCC: Log_10_ of the somatic cell count values; MASTNC: new cases of subclinical mastitis during the experiment; MaxVL: maximum vacuum level; AvgVL: average vacuum level; ns: not significant. Values in the same row with different letters (a, b) differ at *p* < 0.05.

**Table 3:** Experiment developed in mid-lines pipes milking system: results of the variables studied (least square means ± standard error of the mean) according to the different combinations of system vacuum level and pulsation parameters.

Variable	ML-40KPa-90ppm-60/40	ML-40KPa-120ppm-60/40	SEM	SL
Machine Milk Yield (Kg)	1.71	1.75	0.07	ns
Hand-Stripping Milk (g)	186	200	17	ns
Residual Milk (g)	115	99	9	ns
Total Milk (Kg)	2.01	2.04	0.07	ns
Milking Duration (s)	158	154	8	ns
PFR (Kg/min)	1.1	1.1	0.06	ns
AvgFlow (Kg/min)	0.65	0.68	0.03	ns
nDCA(%)	36 (19.9)	39 (21.9)		ns
MD (DCA)	195 a	273 b	22	<0.01
ITWT (%)	48.8	52.2	3.1	ns
ITWA (%)	58.4	58.1	5.6	ns
ITEWA (%)	22.2	22.2	1.7	ns
LogSCC (SCC × 1000)	2.57 (375)	2.41 (256)	0.09	ns
MASTNC	7	5		ns
Fat	5.15	5.11	0.11	ns
Protein	3.22	3.23	0.05	ns
Lactose	4.13	4.17	0.02	ns
Milk Cortisol (ng/mL)	0.44	0.45	0.04	ns
MaxVL (KPa)	39.3	39.3	0.1	ns
AvgVL (KPa)	35.6	35.8	0.3	ns
Vacuum Drop (KPa)	11.2	11.4	1.2	ns

PFR: peak flow rate; AvgFlow: average milk flow; nDCA: frequency of double cluster attachment; MD (DCA): milking duration in case of a double cluster attachment; ITWT: increase in teat wall thickness; ITEWA: increase in teat-end wall area; ITWA: increase in teat wall area; LogSCC: Log10 of the somatic cell count values; MASTNC: new cases of subclinical mastitis during the experiment; MaxVL: maximum vacuum level; AvgVL: average vacuum level; ns: not significant. Values in the same row with different letters (a, b) differ at *p* < 0.05.

## Data Availability

The data presented in this study are available on request from the first author.
